# Asphaltene-derived nanocomposites for the removal of emerging pollutants and its antimicrobial effects: batch and continuous column studies

**DOI:** 10.1007/s11356-024-33049-8

**Published:** 2024-03-26

**Authors:** Abhishek Nayak, Vaishnavi P Karkare, Kapil Sadani, Harshini Dasari, Arumugam Sivasamy, Nethaji Sundarabal

**Affiliations:** 1 Department of Chemical Engineering, Manipal Institute of Technology, Manipal Academy of Higher Education (MAHE), Manipal, Udupi, 576104 Karnataka India; 2 Catalysis Science Laboratory & Cell for Industrial Safety and Risk Analysis (CISRA), CSIR-Central Leather Research Institute Adyar, Chennai, 600020 India; 3 Department of Instrumentation & Control Engineering, Manipal Institute of Technology, Manipal Academy of Higher Education (MAHE), Manipal, Udupi, 576104 Karnataka India

**Keywords:** Emerging pollutants, Antibiotic adsorption, Asphaltenes, Column adsorption, Antimicrobial study, Activated carbon

## Abstract

Emerging contaminants are diverse ecotoxic materials requiring unique treatment for removal. Asphaltenes are environmentally hazardous carbon-rich solid waste product of the petroleum industry. In the current work, asphaltene-derived activated carbon (AC) was loaded with silver (Ag/AC) and used to remove amoxicillin (AMX) and tetracycline (TC) from aqueous phase. The prepared Ag/AC was characterised using FESEM, FTIR, XRD and surface area analysis. The FESEM micrographs confirmed the spherical silver nanoparticle-laden porous AC, and the BET surface area was found to be 213 m^2^/g. Batch adsorption studies were performed, and the equilibrium data were fit into adsorption isotherm and kinetic models. The Ag/AC exhibited superior monolayer adsorption capacity of 1012 mg/g and 770 mg/g for AMX and TC, respectively. The continuous column studies were also performed to evaluate the breakthrough parameters. Furthermore, the antimicrobial activity of the adsorbent was evaluated using zone of inhibition studies. Ag/AC was found to have an 8-mm-diameter zone of microbial inhibition. The obtained results showed that Ag/AC was a promising material for the removal of antibiotics and inhibition of resistance-developed mutated microbes in effluent water.

**Supplementary Information:**

The online version contains supplementary material available at 10.1007/s11356-024-33049-8.

## Introduction

Emerging contaminants are pollutants that require advanced processes for the treatment and removal from the aqueous phase; moreover, their environmental impact is not entirely understood (Gavrilescu et al. 2015). Organisations for monitoring emerging environmental substances, such as the NORMAN database, have identified more than 100,000 emerging pollutants, including personal care products, surfactants, pharmaceutical drugs and fuel additives (Mohammed Taha et al. 2022). Though present in low concentrations, these pollutants are known to cause an imbalance in aquatic life by genotoxic effects and have also been reported to cause cancer, reduced fertility, thyroid imbalance and feminisation among terrestrial organisms (Marcoux et al. 2013; Patel et al. 2020). Recently, pharmaceutical drugs such as antibiotics have been identified in the treated wastewater because of their increased usage as countermeasures for various bacterial infections. The use of antibiotics has been relevant for over five decades, and the fate of these medicines is unclear. Depending on the source of wastewater, these antibiotic pollutants are found in the concentration ranges of ng/L to µg/L in the effluent of sewage treatment plants (AL Falahi et al. 2022). Since the waterbodies are home to many bacteria, the presence of antibiotics in the wastewater plays a significant role in the ineffectiveness of antibiotics by causing antibiotic resistance in target bacteria.

Antibiotic resistance is a global concern and has been described as a ‘major global health challenge’ by the World Health Organization (WHO) (Lucien et al. 2021; Kirchhelle and Podolsky 2022). Often, antibiotic resistance in bacteria is developed due to repeated exposure of target bacteria to antibiotic and endocrine disruptor compounds in contaminated water streams (Weiss et al. 2005; Daghrir and Drogui 2013; Garcia-Rodríguez et al. 2014). Among all the antibiotics, amoxicillin (AMX) and tetracycline (TC) are the most commonly used antibiotics to counter broad-spectrum bacterial infections in human and animal medicine (Daghrir and Drogui 2013). AMX is a penicillin-based broad-spectrum antibiotic widely used in human and veterinary medicine, while TC is a broad-spectrum antibiotic typically used in veterinary medicine and agriculture to counter bacterial infections. Moreover, AMX and TC are widely used drugs, with about 50–80% of the dose unabsorbed by the body. Hence, these antibiotics form emerging pollutants introduced to the environment in the form of human waste, animal waste and pharmaceutical industrial waste. These excreted antibiotics and metabolites ultimately find their way into the wastewater (Saadati et al. 2016). Both AMX and TC are stable compounds; however, they form some hazardous by-products upon degradation. These stable compounds have already led to ineffective countermeasures for bacteria due to continuous unintended exposure of bacteria to these antibiotics in the wastewater. During the exposure, the bacteria mutate to develop genes that are resistant to antibiotics, and this antibiotic resistance proliferates rapidly in the microbial community (MacLean and San Millan 2019). Hence, most of these antibiotics are being replaced with alternative drugs. In addition to antibiotic resistance, TC causes algal growth inhibition disruption in carbon and nitrogen removal from the environment; similarly, AMX causes increased zebrafish mortality. Hence, a removal strategy is necessary to counter the ill effects of antibiotic-polluted water.

Conventional wastewater treatment processes such as sedimentation, filtration, chlorination and ultraviolet radiation are not equipped to remove the emerging micropollutants such as antibiotics (Dharupaneedi et al. 2019). The antibiotic concentration after the conventional water treatment is often observed to be in the range of ng/L, which is sufficient for the microbes to develop antibiotic resistance. Hence, advanced physico-chemical processes such as electro floatation (Jones et al. 2005), Fenton degradation (Bolobajev et al. 2016), photocatalytic degradation (Ai et al. 2018; Balakrishnan et al., 2023), ozonation (Rivas et al. 2011) and adsorption (Francoeur et al. 2023) have been explored for the removal of such pollutants.

Adsorption by activated carbon (AC) is well known for its high removal efficiency and low cost. AC from various sources is studied for the adsorption of antibiotics from wastewater. However, AC derived from industrial wastes such as asphaltenes, a rich source of carbon and heteroatoms, are not studied for the adsorptive removal of antibiotics. Asphaltenes are carbon-rich petroleum waste produced in various stages of petroleum processing (Kamkar and Natale 2021). The asphaltenes pose various operational problems in the crude by increasing viscosity, density, scaling and maintenance cost. Due to these associated problems and lack of commercial value, the asphaltenes are often separated from the crude and discarded into tailing ponds (Xu et al. 2013) and landfills (Minai-Tehrani et al. 2015). Thus, asphaltenes contribute to a significant expenditure in petroleum industries. Moreover, disposal of these asphaltenes poses an environmental hazard (Kokal and Sayegh 1995; Saadi et al. 2023). Therefore, preparing AC from asphaltenes will lead to the valorisation of the solid wastes and also aid in reducing solid waste disposal problems. In addition, AC-derived asphaltenes are highly porous and have higher surface area as compared with most of the natural sources of carbon used for AC (Han et al. 2019) as shown in Table 1. Incorporation of silver nanoparticles over the surface of this high surface area AC derived from asphaltenes (Ag/AC) may contribute to its antimicrobial properties adding to the adsorption property. Hence, the current study signifies the application of asphaltene-derived Ag/AC composites for the removal of antibiotics such as AMX and TC from the aqueous phase.

**Table 1:** Comparison of specific surface area and maximum monolayer adsorption capacity with adsorbents reported in the literature

Source of AC	Specific surface area (m^2^/g)	Q_m_ (mg/g)	Reference
Granular-activated carbon	462	3.16 (AMX)	de Franco et al. (2017)
Durian shell	917	140 (AMX), 146 (TC)	Yazidi et al. (2020)
*Arundo donax* Linn	1065	345 (AMX)	Chayid and Ahmed (2015)
*Prosopis juliflora*	946	714 (AMX)	Chandrasekaran et al. (2020
Eucalyptus sawdust	1263	485 (AMX)	Shi et al. (2023)
Olive stone	1174	57 (AMX)	Limousy et al. (2017)
Apricot nut shell	307	308 (TC)	Marzbali et al. (2016)
Tyre pyrolysis char	814	85 (TC)	Acosta et al. (2016)
Macadamia nut shells	1524	455 (TC)	Martins et al. (2015)
Activated carbon copper sulphate impregnated	581	64.12 (TC)	Costa and Féris (2020)
Lignin	931	475 (TC)	Huang et al. (2014)
Asphaltenes	1800	412 (AMX), 746 (TC)	This work

In this work, asphaltenes are used as the carbon source for the preparation of AC to adsorb AMX and TC, and the AC is impregnated with silver nanoparticles (Ag/AC) to achieve bacterial inhibition. Hence, Ag/AC is utilised for the inhibition of mutated bacteria in addition to the removal of antibiotics. The prepared Ag/AC is studied for removal of AMX and TC from the aqueous solution both in batch and continuous mode of operation. In addition, the antimicrobial property of Ag/AC is studied for the combined effect of removal of antibiotics and microbial inhibition.

## Materials and methods

### Chemicals

n-Heptane (C_7_H_16_, synthesis, Loba Chemie), potassium hydroxide (KOH, Emplura, Sigma-Aldrich), hydrochloric acid (HCl, Extra pure, Loba Chemie), silver nitrate (AgNO_3_, extra pure, Sigma-Aldrich), sodium borohydride (NaBH_4_, > 98%, Sigma-Aldrich), amoxicillin trihydrate (C_16_H_19_N_3_O_5_S.3H_2_O, > 9 8%, TCI Japan, AMX), tetracycline hydrochloride (C_22_H_24_N_2_O_8_.HCl, > 98%, TCI Japan, TC), nutrient broth (NutriSelect plus, Sigma-Aldrich), *E. coli* (*Escherichia coli* B40) and nutrient agar (NutriSelect plus, Sigma-Aldrich) were used in the work. All the chemicals used in this work were used without further processing.

### Extraction of asphaltenes from bitumen

Bitumen was mixed with n-heptane in a 1:40 (w/vol) ratio for 24 h. The solution was filtered on a Whatman filter paper, separating the solid residue. The separated solid was dried, and the dried asphaltenes were stored in an air-tight container (Nassar et al. 2011).

### Preparation of asphaltene-derived activated carbon

The asphaltene-derived AC was prepared with the extracted asphaltenes and KOH as the activating agent. The KOH and asphaltenes were mixed in the ratio 4:1 (w:w) and were ground finely. The mixture was subjected to high-temperature activation at 800 °C for 1 h in a nitrogen atmosphere in the muffle furnace. The sample was then washed with dilute HCl followed by distilled water wash till the pH was neutral. The solid sample was dried in a hot air oven at 80 °C stored in an air-tight container (Shalini et al. 2021).

### Preparation of Ag/AC using asphaltene-derived activated carbon

The prepared AC was impregnated with silver nanoparticles using co-precipitation (Eltugral et al. 2016). A total of 0.5 g prepared AC was added to 0.1 M silver nitrate solution. The solution was stirred for an hour, dispersing the AC. The mixture was reduced using 10 ml 10% NaBH_4_ solution, then stirred for another 2 h. The solid nanocomposite was then separated, washed, dried and stored in a desiccator.

### Characterisation of prepared materials

The surface area and pore volume are important characteristics of an adsorbent. Higher surface area, and pore volume of nanocomposite, provides higher sites for adsorption. BET-specific surface area and pore volume of the prepared Ag/AC were determined by Smart sorb 92/93 instrument using the BET adsorption model. The crystallinity of Ag/AC before and after adsorption of AMX and TC was tested by XRD analysis. Rigaku Miniflex 600 (5th gen) instrument was used where the X-ray generation was established by 40 kV and a current of 15 mA, X-ray generation was in the presence of a nickel filter, and the scanning was done from 5 to 80°. The surface moieties of the prepared Ag/AC were identified using FTIR. FTIR of Ag/AC before and after adsorption of AMX and TC was analysed using Shimadzu-8400S. The surface characteristics such as porosity, particle size and impregnation were studied by FESEM imaging, and elemental confirmation of the prepared adsorbent before and after adsorption was determined by EDX analysis carried out in Carl Zeiss ULTRA 55 instrument.

### Batch adsorption studies

The adsorption studies were performed for two antibiotics, AMX and TC, using asphaltene-derived Ag/AC nanocomposite. The antibiotic solutions of AMX and TC were prepared in double distilled water, and studies on the effect of pH, adsorbent dosage, adsorbate concentration and kinetic studies were carried out. The effect of pH on adsorption was studied, with the pH of the antibiotic solution varying from 2 to 12, while antibiotic concentration was maintained at 50 mg/L and adsorbent dosage fixed at 0.5 g/L. Similarly, the effect of adsorbent dosage studies included the variation of adsorbent dosage from 0.05 to 0.5 g/L in antibiotic solution concentration fixed at 50 mg/L. The effect of initial antibiotic concentration was carried out with a 1.25 g/L adsorbent dosage, while initial antibiotic concentration varied from 100 to 1000 mg/L. All the batch adsorption studies were performed for 24 h at 30 °C.

The batch studies data was further analysed using isotherm model to describe adsorbent-adsorbate reaction. The equilibrium data was analysed using the Langmuir model (Nebaghe et al. 2016) and Freundlich adsorption isotherm model (Parimal et al. 2010) as mentioned in Eqs.1 and 2, respectively. The Langmuir isotherm is governed by the assumption that the adsorption process is monolayer and the adsorption sites are homogenous (Muthamilselvi et al. 2016); whereas Freundlich isotherm represents multilayer adsorption on heterogenous adsorption sites.

1 $$Q=\frac{{Q}_{{\text{m}}} { K}_{{\text{L}}} C}{(1+ { K}_{{\text{L}}} C)}$$

2 $$Q={K}_{{\text{F}}} {C}^{1/n}$$

where *Q* is equilibrium adsorption capacity, *Q*_m_ is the maximum monolayer adsorption capacity, *C* is the concentration of antibiotics at equilibrium (mg/g), *K*_L_ is Langmuir equilibrium constant (L/mg), *K*_F_ is the Freundlich adsorption coefficient which represents the adhesion ability of the adsorbate onto the adsorbent and *n* is the heterogeneity factor.

The adsorption kinetics was carried out for 500 mg/L initial concentration of antibiotic solution with the adsorbent dosage of 1.25 g/L. The kinetic data was further analysed using pseudo-first-order (PFO) (Li et al. 2011) and pseudo-second-order (PSO) (Revellame et al. 2020) kinetic models, as given in Eqs. 3 and 4, respectively. The PFO model is described for the adsorption process limited by physisorption where the rate is proportional to adsorption sites, while PSO best explains the irreversible chemisorption where the rate of adsorption is a function of adsorption capacity of the material.

3 $${\text{ln}}\left({Q}_{{\text{e}}}-{Q}_{{\text{t}}}\right)={\text{ln}}\left({Q}_{{\text{e}}}\right)-{k}_{1} t$$

4 $$\frac{t}{{Q}_{{\text{t}}}}=\frac{1}{{k}_{2} {Q}_{{\text{e}}}^{2}}+\frac{t}{{Q}_{{\text{e}}}}$$

where *Q*_e_ is the equilibrium adsorption capacity (mg/g), *Q*_t_ is the adsorption capacity at time ‘*t*’ (mg/g) and *k*_1_ and k_2_ are PFO and PSO rate constants, respectively.

Furthermore, to understand the mechanism and the rate-limiting steps of adsorption, the Weber-Morris intraparticle diffusion model (Zahoor and Mahramanlioglu 2011) and Boyd model (Yao and Chen 2017) were used in the form of Eqs. 5 and 6.

5 $${Q}_{{\text{t}}}={k}_{{\text{i}}} \sqrt{{\text{t}}}+C$$

6 $${B}_{{\text{t}}}=-0.4977-{\text{ln}}(1-F)$$

where *Q*_t_ is the adsorption capacity at time ‘*t*’ (mg/g), *k*_i_ is intraparticle diffusion rate constant (mg/g min^1/2^) and *F* (dimensionless) is fractional adsorption capacity.

### Continuous adsorption studies of AMX and TC using Ag/AC

The fixed bed column was fabricated with a bed diameter of 1.5 cm and a bed height of 1 cm. The column was packed with the prepared Ag/AC for treating the antibiotic-laden aqueous solution. The coarse and fine glass beads were packed on top and bottom of the nanocomposite bed to keep the bed intact. In total, 500 mg/L antibiotic solutions were continuously fed from the top until the bed was exhausted. The samples were collected at regular intervals and analysed for the concentration of the antibiotics. The fixed bed parameters effluent volume (*V*_eff_), breakthrough time (*t*_b_), bed exhaustion time (*t*_e_) and adsorption percentage were calculated from the experimental data using the following equations (Atar et al. 2011; Muthamilselvi et al. 2018)

7 $${{\text{V}}}_{{\text{eff}}}=F {{\text{t}}}_{{\text{total}}}$$

*F* is the feed flow rate, and *t*_total_ is the total flow time.

Similarly, the total amount of antibiotics passed through the column was calculated using the formula,

8 $${m}_{{\text{total}}}={C}_{0} F {t}_{{\text{e}}}$$

where *C*_0_ is the initial antibiotic concentration.

Finally, the total antibiotic removal percentage was calculated using,

9 $$\mathrm{Percentage\;removal }\left(\mathrm{\%}\right)=\frac{{m}_{{\text{ads}}}}{{m}_{{\text{total}}}}\times 100$$

where the total adsorbate adsorbed in the column (*m*_ads_) was calculated from flowrate multiplied by the area above the breakthrough curve.

10 $${m}_{{\text{ads}}}=F A$$

where *A* is the area above the breakthrough curve.

### Residual concentration analysis of AMX and TC

The residual concentration from each study was estimated using Shimadzu/UV 1800 Series UV Visible spectrophotometer. The estimation was carried out at 227.2 nm for AMX (Moradi et al. 2022) and 356 nm for TC (Ren et al. 2017) with distilled water as a reference. The adsorbent was separated from the residual solution using centrifugation.

### Microbial inhibition studies of the prepared Ag/AC

The prepared asphaltene-derived AC and Ag/AC were tested for their antimicrobial activity. The antimicrobial activity was analysed using a zone of inhibition test using the disk diffusion method. The *E. coli* culture was spread on the nutrient, and the prepared material-coated disk was placed on it and incubated at 35 °C for 18 h. The inhibition zone was then determined from the clear area around the disk, and the diameter of the circular area was the zone of inhibition (Matuschek et al. 2014).

## Results and discussions

### Characterisation of prepared material

**Fig. 1:**
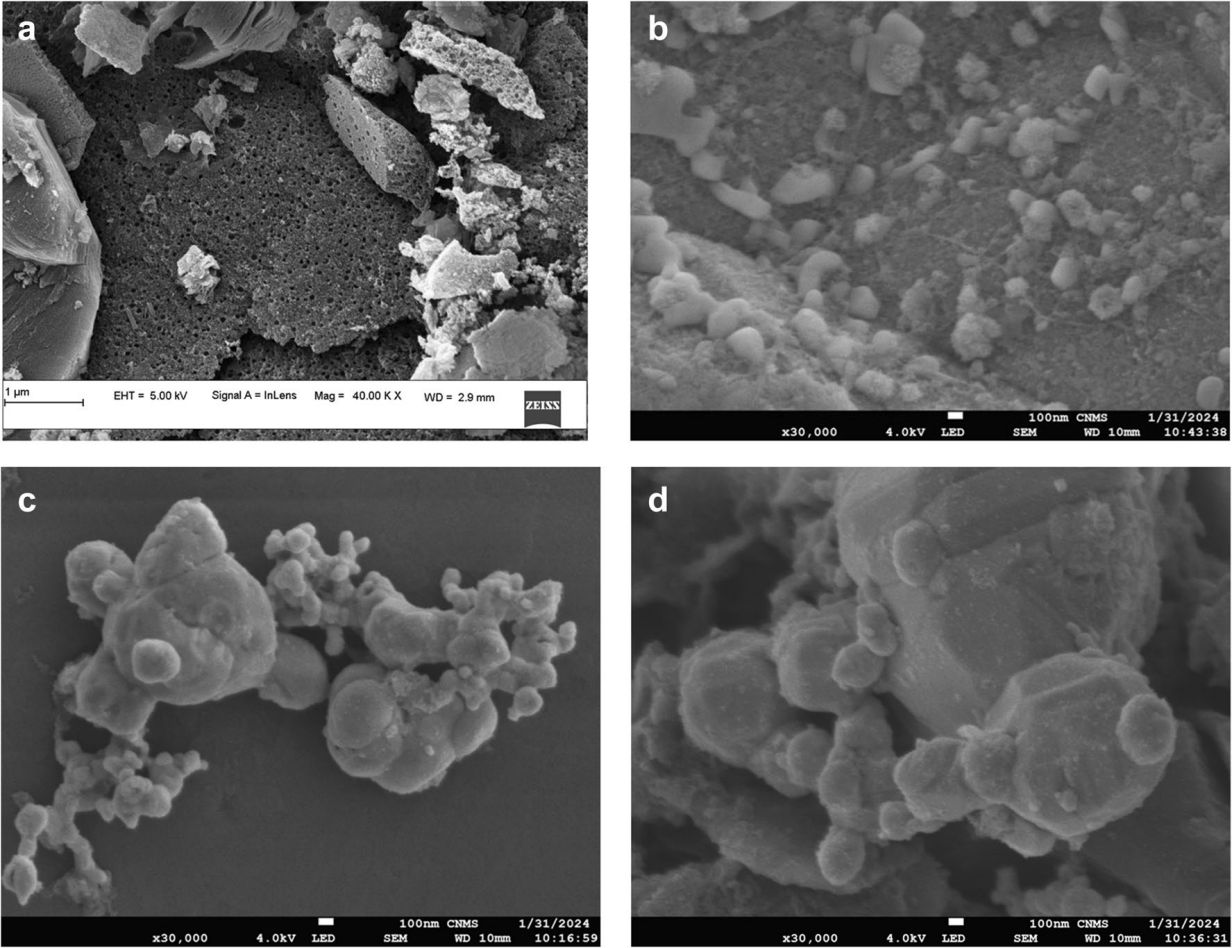
FESEM micrographs of the prepared asphaltene-derived **a** AC, **b** Ag/AC, **c** Ag/AC after AMX adsorption and **d** Ag/AC after TC adsorption

The prepared asphaltene-derived nanocomposite was characterised using FESEM, EDX, BET, XRD and FTIR. Figures 1a and b depict the FESEM micrograph of the asphaltene-derived AC and Ag/AC, respectively. As an activating agent in the preparation of asphaltene-derived AC, the KOH influenced imparting pores by pyrolytic decomposition. The uniform macropores on the AC were confirmed from the FESEM micrographs. Further, from Fig. 1b, the FESEM micrographs confirmed the spherical silver nanoparticles impregnated onto the porous activated carbon. Figure 1c and d represents Ag/AC after adsorption of AMX and TC respectively. The presence of agglomerated adsorbate molecules along with the silver nanoparticles could be observed. From the EDX in Fig. S1a, the presence of carbon and silver in Ag/AC before adsorption was confirmed. However, from the EDX of Ag/AC after adsorption of AMX in Fig. S1b and S1c, traces of sulphur, oxygen and nitrogen were observed along with carbon and silver, indicating the presence of AMX. Similarly, in the case of Ag/AC after adsorption of TC, trace quantities of nitrogen and oxygen were observed in addition to carbon and silver, which confirmed the presence of the adsorbed TC molecules. In addition, the prepared Ag/AC was characterised for surface area and pore volume using BET surface area analyser. From the BET analysis, the bare AC had 1800 m^2^/g and 1.8 cm^3^/g, surface area and pore volume, respectively, and the surface area of AC after Ag impregnation was 213 m^2^/g, and the pore volume was 0.12 cm^3^/g.

**Fig. 2:**
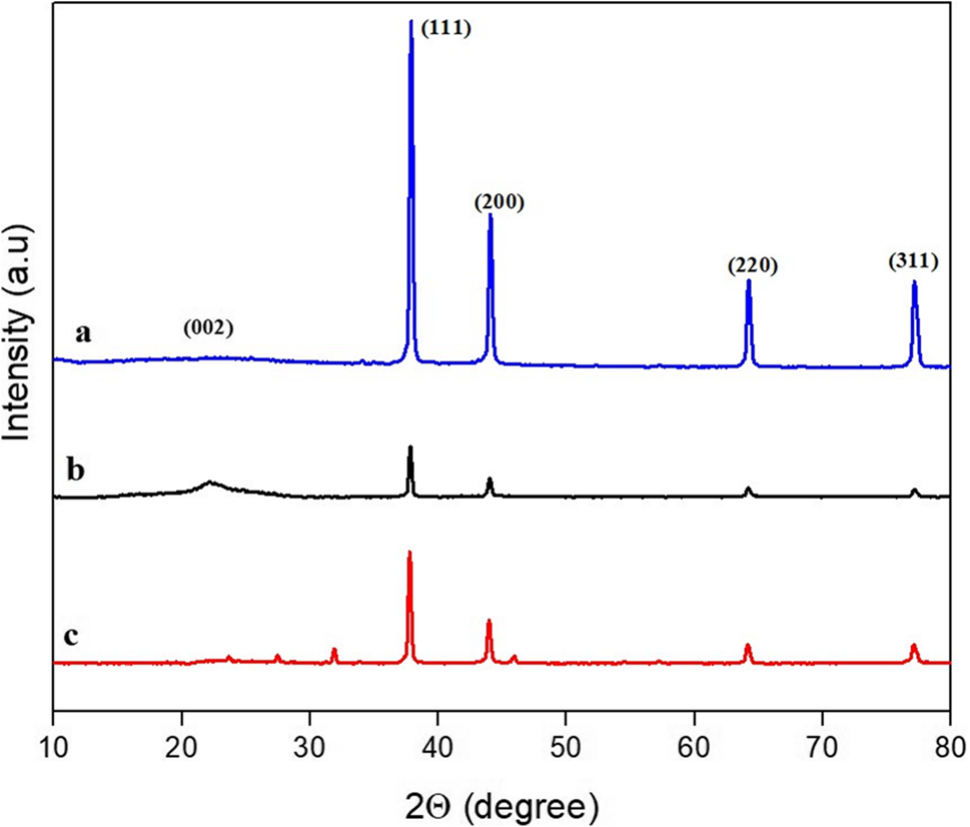
XRD pattern of asphaltene-derived **a** Ag/AC, **b** Ag/AC after AMX adsorption and **c** Ag/AC after TC adsorption

Figure 2a depicts the XRD of asphaltene-derived Ag/AC. The broad peak extending from 15–30° 2Theta confirms the presence of AC from its characteristic (002) peak. The XRD patterns of the Ag/AC also had the (002) distinct peak along with (111), (200), (220) and (311) representing silver, thereby confirming the prepared Ag/AC nanocomposite (JCPDS card: number 04–0783) (Sarkar and Das 2019). The distinct peaks representing carbon and Ag in the XRD are indications of Ag deposition onto AC. Figures 2 b and c represent the XRD of Ag/AC after adsorption of AMX and TC, respectively. The XRD spectrum in Fig. 2b and c had no significant variations from pristine Ag/AC, indicating that Ag/AC did not undergo any structural changes during adsorption (Costa and Féris 2020).

**Fig. 3:**
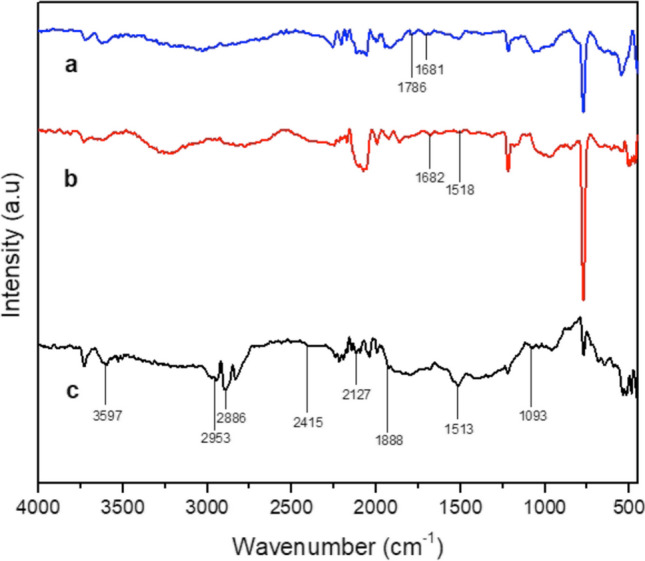
FTIR spectrum of prepared asphaltene-derived **a** Ag/AC after AMX adsorption, **b**) Ag/AC after TC adsorption and **c** Ag/AC

FTIR was carried out for prepared Ag/AC, Ag/AC after adsorption of AMX and TC, and the spectrum is shown in Fig. 3. From Fig. 3c, the peaks representing C = N, C = C and C–O were observed at 2127 cm^−1^, 2415 cm^−1^ and 1093 cm^−1^. Symmetric stretching of the CH_2_ was observed at 2886 cm^−1^; symmetric and asymmetric bending vibrations of C–H, C = O of carboxylic acid and peaks representing aliphatic carbon were observed at 1513 cm^−1^, 1888 cm^−1^ and 2953 cm^−1^, respectively. In addition, peaks related to the O–H stretching of water molecules were identified at 3597 cm^−1^ (Cuhadaroglu and Uygun 2008; Chandra Joshi et al. 2022). Peaks at 1786 and 1681 cm^−1^ representing C = O in O = C–N and O = C–OH of AMX from AMX-adsorbed Ag/AC were observed in Fig. 3a (Zha et al. 2013). Similarly, bending vibrations of C = O and –N–H_2_ amide groups of TC from TC-adsorbed Ag/AC were observed at 1518 and 1682 cm^−1^ in Fig. 3b (Martins et al. 2015).

### Batch adsorption studies of the AMX and TC adsorbed onto Ag/AC

The prepared Ag/AC nanocomposite was used as an adsorbent in AMX and TC adsorption studies. The batch adsorption studies included the effect of pH, the effect of adsorbent dosage and the effect of initial antibiotic concentration. The batch adsorption studies were carried out with pH ranging from 2 to 12, as shown in Fig. S2. The pH did not affect the AMX adsorption when maintained below pH 10. However, further increment of the pH had a negative impact on adsorption because of the deprotonation of AMX and repulsion of adsorbate-adsorbent. Hence, further studies were conducted at a pH of 6 (Bezerra et al. 2013). Similarly, the pH had an insignificant effect on the adsorption of TC onto Ag/AC. Adsorption of TC mostly remained unaffected with the increase of pH from 2 to 6. However, the pH had adverse effects when it was increased beyond 6; the adsorption decreased as the pH varied above 6, indicating unfavourable conditions. TC is found to form cationic groups under acidic conditions around pH 3.3, zwitter ions from 3.3 to 7.7 and anionic form at a pH range greater than 7.7 (Wang et al. 2008). The TC is mainly found to be neutral or slightly negatively charged at the neutral pH, which may have enhanced the interaction between the prepared adsorbents, thereby increasing the extent of adsorption.

The effect of dosage on the adsorption of AMX and TC was carried out by varying the dosage from 0.05 to 0.5 g/L, as shown in Fig. S3. The percentage removal of antibiotics increased slightly with the adsorbent dosage from 0.05 to 0.5 g/L, while the adsorption capacity reduced drastically. The maximum adsorption removal of antibiotics was found with a 0.4 g/L dosage.

The effect of initial antibiotic concentration on adsorption onto the adsorbate was studied by varying the initial concentration from 100 to 1000 mg/L. It was observed that the percentage adsorption decreased with the increase of the initial concentration of AMX. However, the adsorption capacity of the adsorbent increased with increasing initial concentration and saturation towards 1000 mg/L, suggesting the saturation of the adsorbent surfaces. Similar observations were made for TC adsorption onto Ag/AC.

**Fig. 4:**
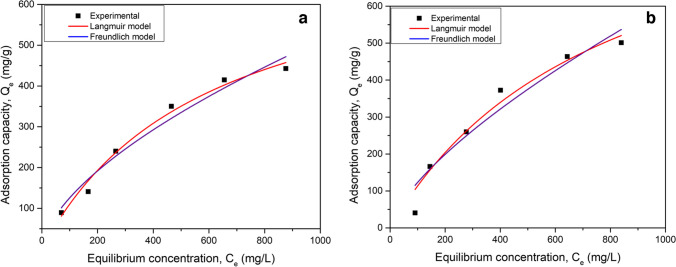
Langmuir and Freundlich isotherm model plots for the adsorption of **a** AMX and **b** TC, onto Ag/AC

The adsorption data were studied using Langmuir and Freundlich adsorption isotherm models. Figures 4a and b represent Langmuir and Freundlich isotherm models fitting for adsorption of AMX and TC, respectively. It could be observed that Langmuir isotherm model is closer to the experimental data points when compared to the Freundlich isotherm model. Similarly, for TC, adsorption data was used in the isotherm studies, and it was observed to follow the Langmuir isotherm model for adsorption. Hence, monolayer adsorption was observed for both TC and AMX. The Langmuir and Freundlich isotherm parameters for the adsorption of AMX and TC onto Ag/AC are shown in Table 2. The maximum monolayer adsorption capacity (*Q*_m_) was calculated using the Langmuir isotherm model and was 1012 mg/g and 770 mg/g for AMX and TC, respectively. Similarly, the maximum monolayer adsorption capacity was determined for asphaltene-derived AC using a Langmuir isotherm model. *Q*_m_ was observed to be 412 and 746 mg/g for AMX and TC, respectively, indicating the improved adsorption in Ag/AC compared to bare AC. The increase in *Q*_m_ of Ag/AC in comparison to AC may be due to the electropositive charge developed by Ag/AC because of Ag(I). AMX and TC exist in negatively charged form in neutral pH. This negative charge of antibiotics and electropositive charge gained by Ag/AC promotes the adsorption of AMX and TC (Sonal et al. 2020).

**Table 2:** Isotherm and kinetics model parameters of AMX and TC adsorption using Ag/AC

Adsorption models	Model parameters	Ag/AC-AMX	Ag/AC-TC
Langmuir isotherm model	*K*_L_ (L/mg)	0.0012	0.0016
	*Q*_m_ (mg/g)	1012.91	770.14
	*r*^2^	0.95	0.98
Freundlich isotherm model	*K*_F_ (mg/g)	5.07	7.57
	*n*	1.44	1.63
	*r*^2^	0.92	0.96
PFO kinetics model	*Q*_e_, (mg/g)	28.55	42.76
	*k*_1_, (h^−1^)	0.014	0.017
	*r*^2^	0.58	0.71
PSO kinetics model	*Q*_e_ (mg/g)	65.61	78.92
	*k*_2_ (g/mg h)	0.067	0.025
	*r*^2^	0.98	0.99

### Adsorption kinetic studies of AMX and TC adsorbed onto Ag/AC

Adsorption kinetics experiments were carried out for the adsorption of antibiotics onto Ag/AC. Kinetic studies were carried out to study the adsorption kinetics, as shown in Fig. S4a and S4b. The adsorbent dosage was fixed at 1.25 g/L. The kinetic data was tested with linear pseudo-first-order (PFO) and linear pseudo-second-order (PSO) kinetic models. From Fig. S4a and Fig. S4b, it was found that the PSO kinetic model was the best fit for AMX and TC onto Ag/AC. The kinetic model fitting suggests a possible chemical interaction between adsorbate and adsorbent (Lu et al. 2013), possibly from the deprotonated AMX leading to electrostatic interaction, hydrogen bonding, electron conjugates or pi–pi interaction. Similarly, a chemical interaction could also be suggested for the TC adsorption onto Ag/AC. The possible mechanism could be the ionic forms of dimethylammonium, tricarbonyl amide and phenolic diketone functional groups interacting with the adsorbent surfaces. The PFO and PSO model parameters and calculated adsorption capacity are tabulated in Table 2. From Table 2, the adsorption capacity calculated from the model agreed with the experimental value in the case of PSO.

**Fig. 5:**
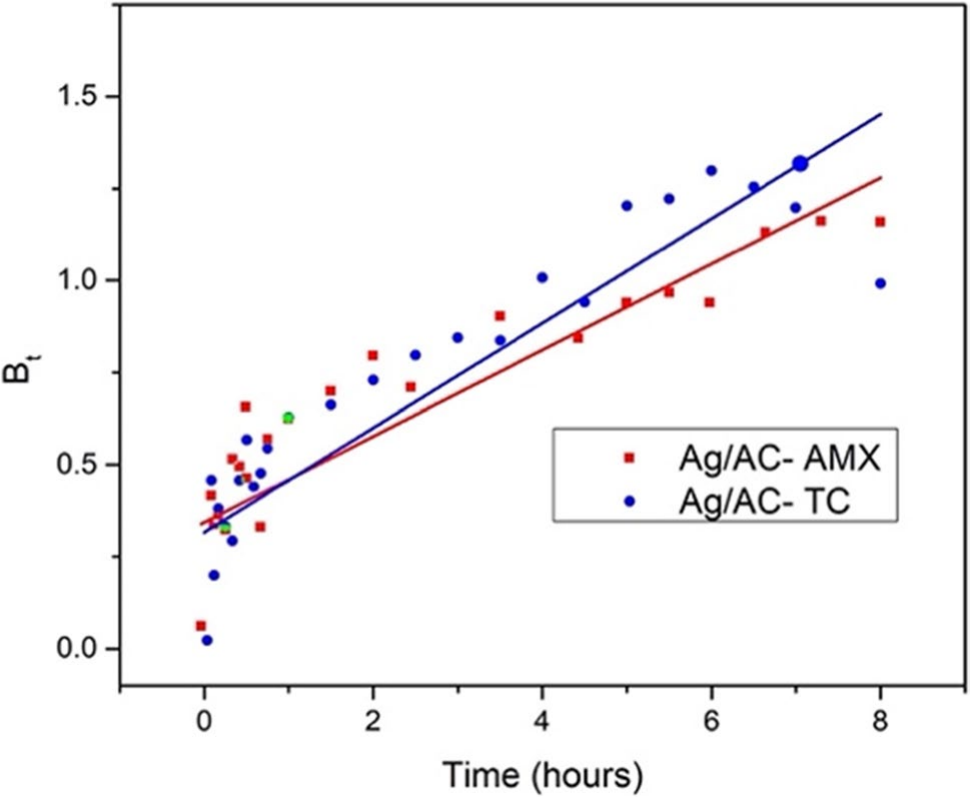
Boyd plot of AMX and TC adsorption using Ag/AC

The intraparticle diffusion model was applied to the adsorption kinetics data to find the rate-limiting step, as shown in Fig. S5. The transport mechanism of the adsorbate leading to adsorption onto the adsorbent was described using this model. The adsorption mechanism could be described using three zones from Fig. S5. The first zone represents the boundary layer diffusion defined by the first linear line extending from the origin. This process was less than 10 min for both AMX and TC adsorption onto Ag/AC. During the process, the antibiotic molecules are driven towards the surface of the adsorbent due to adsorbent-adsorbate interaction. The second zone is described by the curve following the boundary layer diffusion. The zone is governed by an intraparticle diffusion process where the antibiotic molecules enter the pores. This zone was the slowest process in both cases, where the zone for AMX adsorption extended for 6.5 h, and the zone for TC extended for about 7.3 h. Finally, the third zone represents the equilibrium stage of the process during which the process attains equilibrium, represented by a horizontal straight line extending from the end of the intraparticle diffusion zone. The Boyd plot further explained the specific rate-limiting step in the adsorption process. From Fig. 5, a linear curve approaching the origin could be observed for the adsorption of AMX and TC onto Ag/AC, indicating the process was governed by intraparticle diffusion, which was the slowest step (Hu et al. 2011).

### Adsorption mechanism of AMX and TC onto Ag/AC

The isoelectric point (PI) of AMX and TC are 4.7 (Chuo et al. 2014) and 5.4 (Horká et al. 2014), respectively. AMX and TC exist as AMX^+^ and TC^+^ when the solution pH is below PI; similarly, AMX and TC exist as AMX^−^ and TC^−^ in solution pH above PI. From the effect of pH studies, both AMX and TC had higher adsorption affinity around PI, which decreased gradually with an increase in the pH until 8. However, below PI, the sorption was marginally higher, indicating favourable conditions for adsorption. In addition, the prepared Ag/AC may exhibit electropositive behaviour due to the impregnation of Ag(I) which contributes to the higher electrostatic interaction. Hence, the adsorption of AMX and TC on Ag/AC could be attributed to electrostatic interaction of electronegative AMX and TC with electropositive Ag/AC (Sonal et al. 2020).

### Adsorbent reusability studies for AMX and TC adsorption onto Ag/AC

Adsorbent reusability studies were carried out with spent Ag/AC after adsorption of AMX and TC. The study was performed by regenerating the spent Ag/AC using absolute ethanol. In total, 0.5 g/L adsorbent was equilibrated with 200 mg/L of the antibiotics in the aqueous phase for 24 h. The adsorbent was then centrifuged and regenerated with ethanol. The regenerated adsorbent was again used for sorption studies, and the same procedure was carried out for three cycles. From the reusability studies, Ag/AC was found to remove AMX with 97% adsorption efficiency, whereas 95% removal efficiency was observed for TC after three cycles of operation. The reusability studies suggested a minimum loss of efficiency of the prepared Ag/AC over successive regeneration cycle indicating superior reusability of prepared Ag/AC.

### Fixed bed column adsorption studies of AMX and TC adsorbed onto Ag/AC

The fixed bed column adsorption studies were performed with column packing of 0.5 g of adsorbent, the antibiotic concentration was maintained at 500 mg/L and the flow rate of the antibiotic solution was maintained at 3 mL/min. Table 3 depicts the computed column parameters such as effluent volume (*V*_eff_), breakthrough time (*t*_b_), bed exhaustion time (*t*_e_) and adsorption percentage. Effluent volume (*V*_eff_) is the volume of antibiotic solution treated till the bed exhaustion. Similarly, the time at which 1 mg/L antibiotic is observed in the effluent is breakthrough time and the time at which the 99.5% antibiotics is found in the effluent is called bed exhaustion time (*t*_e_). Furthermore, *m*_ad_ is the mass of antibiotic removed by the column, and *m*_total_ is the mass of antibiotic fed to the column.

**Table 3:** Column breakthrough parameters of AMX and TC adsorption using Ag/AC

Adsorbents	*t*_b_ (min)	*t*_e_ (min)	*m*_ad_ (mg)	*m*_tot_ (mg)	*V*_eff_ (L)	% adsorption
Ag/AC-TC	72	190	209.76	285	0.75	73.6
Ag/AC-AMX	74	375	530.43	562.5	1.5	94.3

**Fig. 6:**
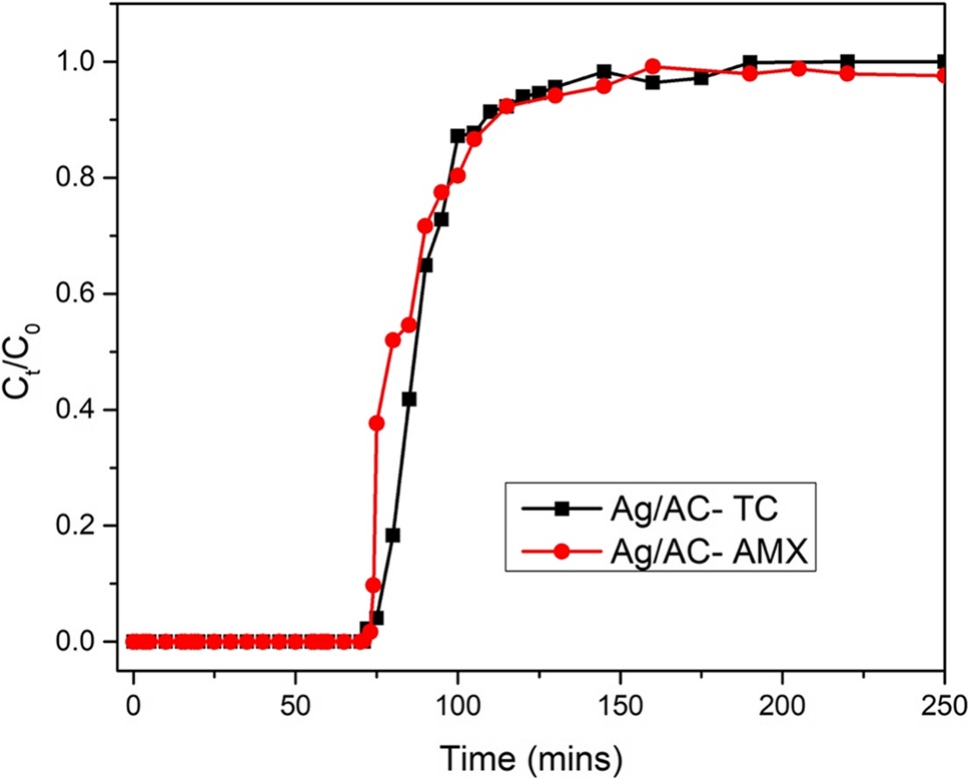
Breakthrough curve for continuous column studies of AMX, TC adsorption using Ag/AC

The column adsorption studies and resulting breakthrough curves are shown in Fig. 6, and the breakthrough parameters calculated are tabulated in Table 3. The adsorption of AMX using Ag/AC was marginally superior to TC, with a breakthrough time of 74 and 72 min, respectively. However, in the case of AMX, the bed exhaustion time (*t*_e_ = 375 min) and total effluent volume treated (*V*_eff_ = 1.5 L) were twice the value of TC (*t*_e_ = 190 min, *V*_eff_ = 0.75 L). Lastly, the prepared Ag/AC had 94.3% and 73.6% adsorption removal of AMX and TC, indicating a higher affinity towards AMX. Ag/AC had a marginally higher breakthrough time for AMX adsorption, indicating higher removal capacity of Ag/AC towards AMX. Similarly, higher exhaustion time and higher effluent volume indicate the higher affinity of Ag/AC towards AMX.

### Antimicrobial studies of the prepared Ag/AC

**Fig. 7:**
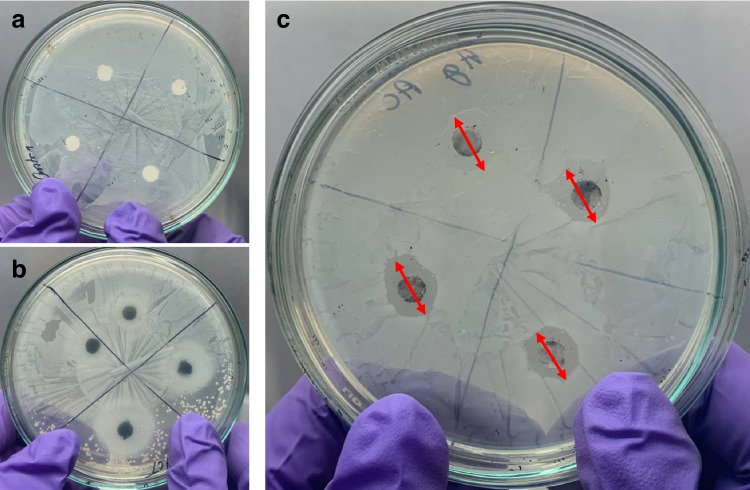
Zone of inhibition studies for **a** control disk, **b** activated carbon-coated disk and **c** Ag/AC-coated disk

The antimicrobial activity of the prepared Ag/AC was determined using the zone of inhibition test. The prepared materials were subjected to the disk diffusion method of the zone of inhibition test, where the nanocomposite materials were coated onto a circular disk and placed in the *E. coli* spread agar plates. The final inhibition zones were compared among the control AC and Ag/AC after 18-h incubation to determine the efficiency of Ag/AC in antimicrobial activity. From Fig. 7, the prepared AC had no visible zone of inhibition, which was anticipated. However, the prepared Ag/AC had an 8-mm zone with no visible *E. coli* growth, suggesting a bacterial inhibition zone. The 8-mm zone of microbial inhibition indicates the area around Ag/AC where the microbes do not grow. This property of Ag/AC is essential for eliminating mutated microbes during the removal process in both batch and continuous modes of operation. The silver nanoparticle is reported to be among the best materials for antimicrobial activity. The antimicrobial process involves the breakage of the outer membrane of microbes, thereby exposing the cellular materials and deactivating respiratory chain dehydrogenases by entering the inner membrane (Tang and Zheng 2018). Hence, silver coating onto activated carbon imparts antimicrobial properties, thereby increasing the efficiency of water treatment.

## Conclusions

Asphaltene-derived AC was impregnated with silver nanoparticles to remove AMX and TC. In this work, AC was prepared from asphaltenes by the KOH activation process and impregnated with silver nanoparticles using co-precipitation. The Ag/AC had both the inherent adsorption property of porous AC and the antimicrobial property of Ag nanoparticles, thereby making it an excellent material for wastewater treatment. The batch adsorption studies included the effect of pH, the effect of adsorbent dosage, isotherm and kinetic studies. There were no significant influence of aqueous phase pH ranging from 2 to 6, but increasing pH beyond 6 drastically affected the studied adsorption process. Further, the batch adsorption studies were governed by the Langmuir adsorption isotherm model and the PSO adsorption kinetic model. The prepared Ag/AC was effective in removal, showing high adsorption capacity towards AMX. In addition, the maximum monolayer adsorption capacity calculated for the adsorbent was 1012 mg/g for AMX and 770 mg/g for TC, which was significantly higher than most reported materials. Further, adsorbent reusability study indicated a minimum loss of adsorption efficiency in Ag/AC over 3 cycles. From fixed bed studies, bed exhaustion time of 375 min and 190 min for 500 mg/L of AMX and TC was observed, indicating an excellent adsorption efficiency, thereby showing a promising future for asphaltene-derived nanocomposite in effluent treatment plants. Furthermore, the antimicrobial studies showed an 8-mm-diameter zone of inhibition of Ag/AC towards *E. coli* growth, suggesting a successful antimicrobial effect. Hence, the prepared asphaltene-derived adsorbent exhibited superior adsorption capacity and antimicrobial properties in simulated aqueous solution. Moreover, the usage of Ag-impregnated asphaltene-derived AC for antibiotic adsorption has advantages of microbial inhibition in addition to adsorptive removal. Hence, these Ag/AC can be further studied for its application in real effluent with complex matrix.

## Supplementary Information

Below is the link to the electronic supplementary material.

Supplementary file1 (DOCX 5974 KB)

## Data Availability

All the required data are provided in the manuscript and supplementary information.

## Code availability

Not applicable for the current study.
