# Supplementary material for: Asphaltene-derived nanocomposites for the removal of emerging pollutants and its antimicrobial effects: batch and continuous column studies
Source: Environ Sci Pollut Res Int. 2024 Mar 26;32(49):28196–208. doi: 10.1007/s11356-024-33049-8 (PMC12712076; doi:10.1007/s11356-024-33049-8)
Supplement: Supplementary file 1 — Supplementary file1 (DOCX 5974 KB) [file 11356_2024_33049_MOESM1_ESM.docx]

**Supplementary Information**

**Asphaltenes derived nanocomposites for the removal of emerging pollutants and its antimicrobial effects: Batch and continuous column studies**

Abhishek Nayak^1^, Vaishnavi P Karkare^1^, Kapil Sadani^1^, Harshini Dasari^1^, Arumugam Sivasamy^2^, Nethaji Sundarabal^1^*

^1*^ Department of Chemical Engineering, Manipal Institute of Technology, Manipal Academy of Higher Education, Manipal, Udupi, Karnataka, India-576104

^2^ Catalysis Science Laboratory & Cell for Industrial Safety and Risk Analysis (CISRA) CSIR-Central Leather Research Institute Adyar, Chennai, India-600020

*Nethaji Sundarabal

Department of Chemical Engineering, Manipal Institute of Technology, Manipal Academy of Higher Education, Manipal, Karnataka, India – 576104

E-mail: nethaji.s@manipal.edu

Phone: 0820 2924316.


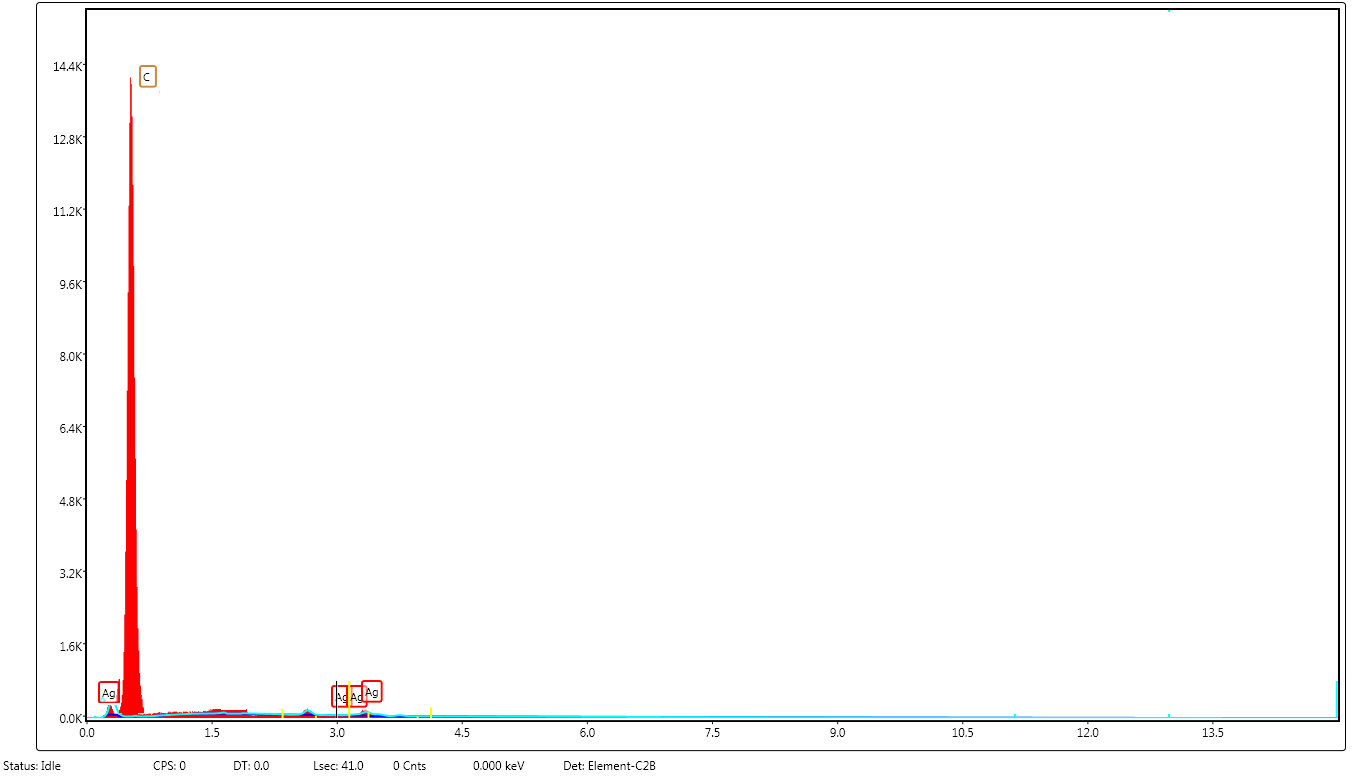


a

**Fig.S1a** EDX spectrum of the prepared Asphaltenes derived Ag/AC before adsorption


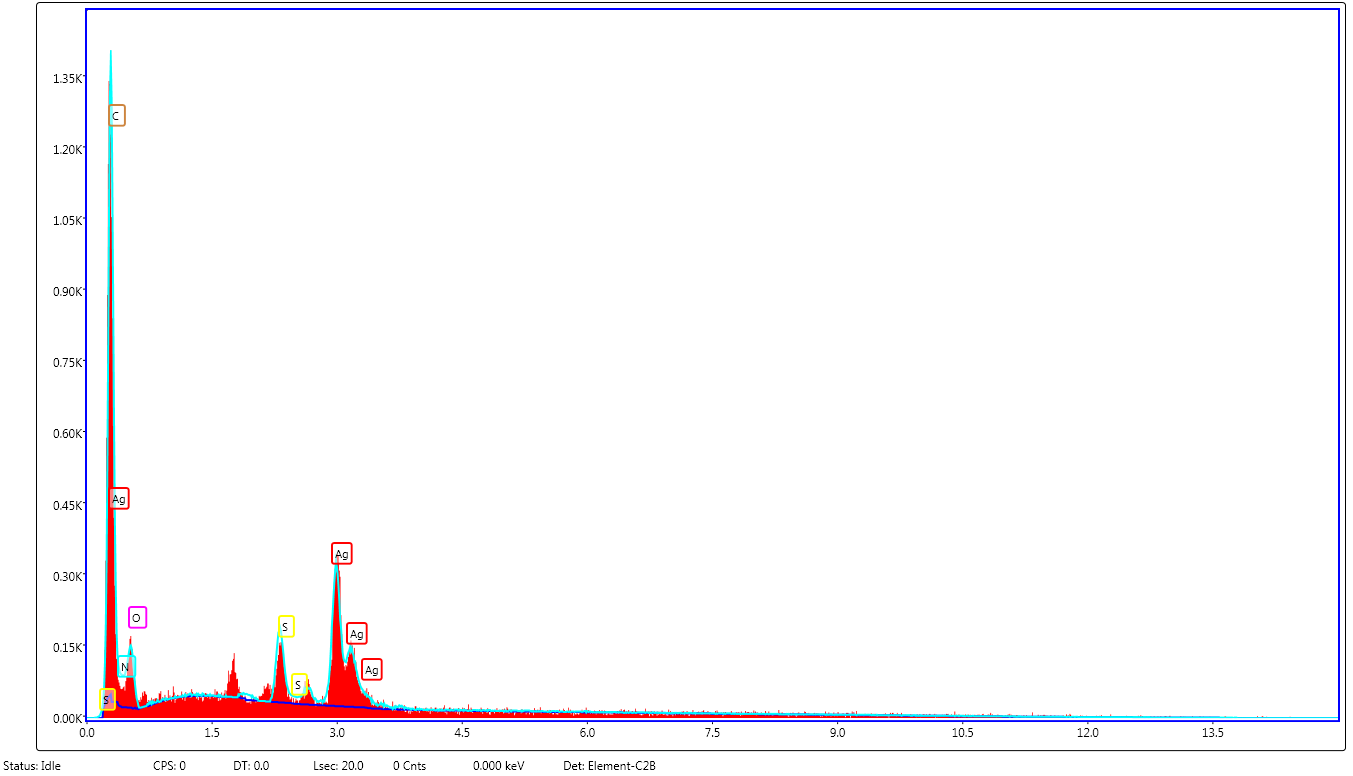


b

**Fig.S1b** EDX spectrum of the prepared Asphaltenes derived Ag/AC after AMX adsorption


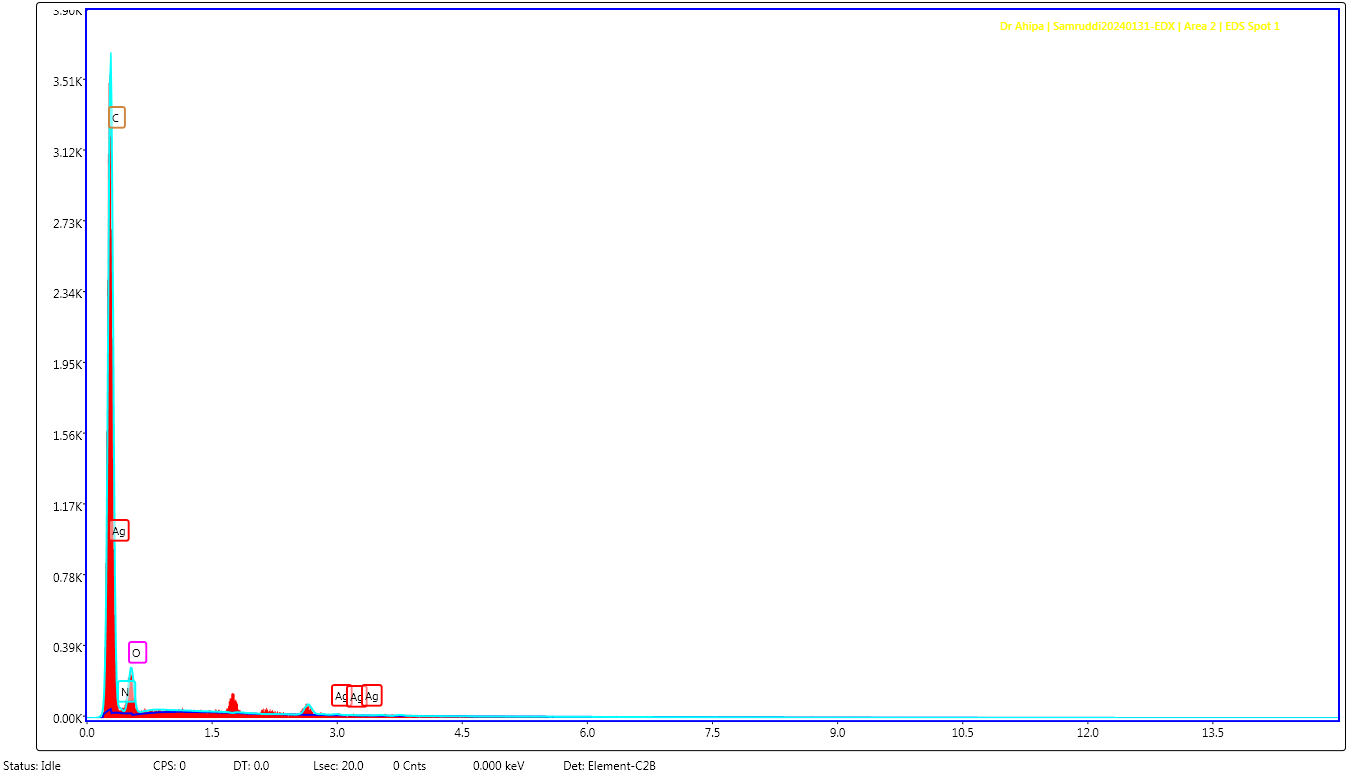


c

**Fig.S1c** EDX spectrum of the prepared Asphaltenes derived Ag/AC after TC adsorption


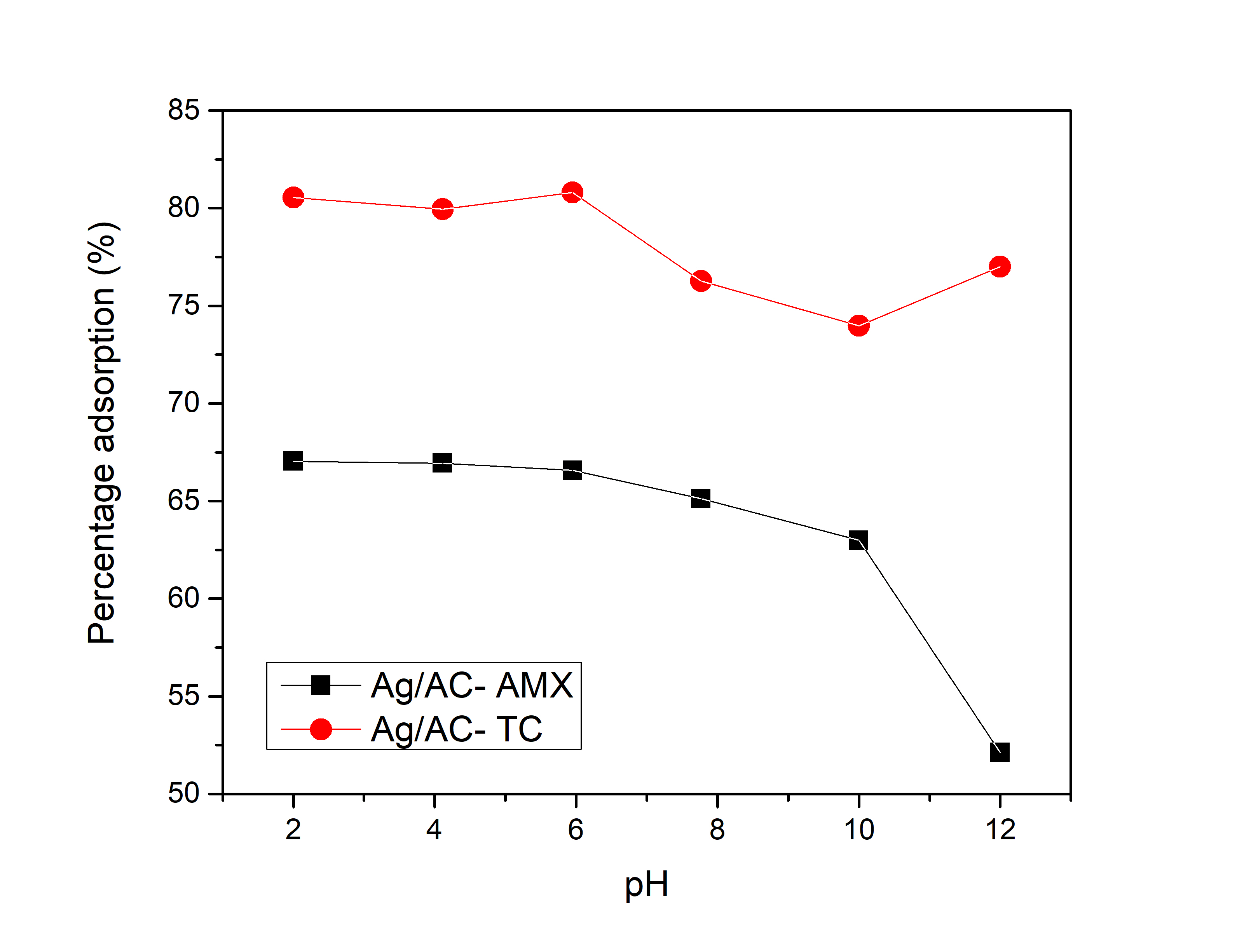


**Fig.S2** Effect of pH on the adsorption of AMX and TC onto Ag/AC


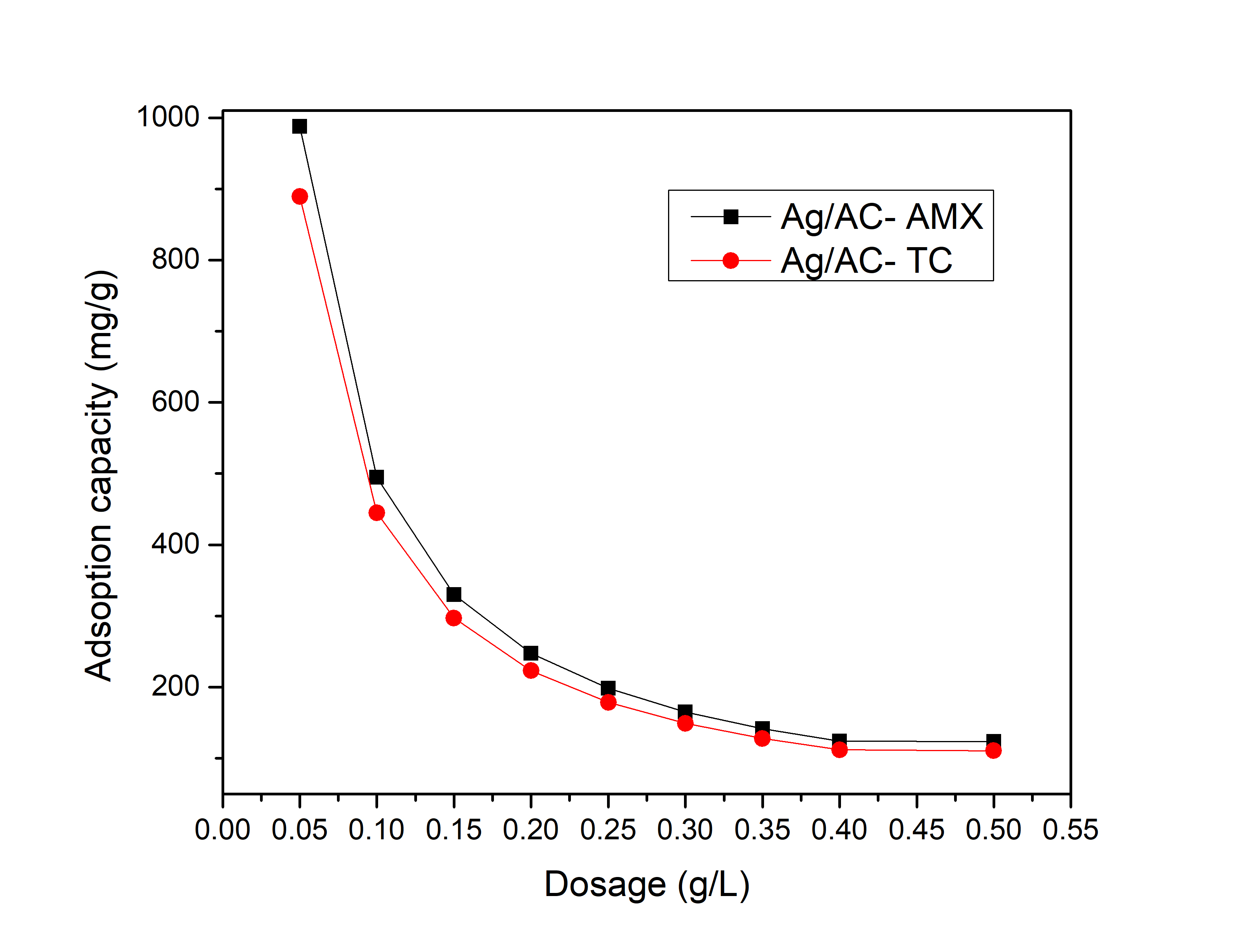


**Fig.S3** Effect of adsorbent dosage on adsorption of AMX and TC onto Ag/AC


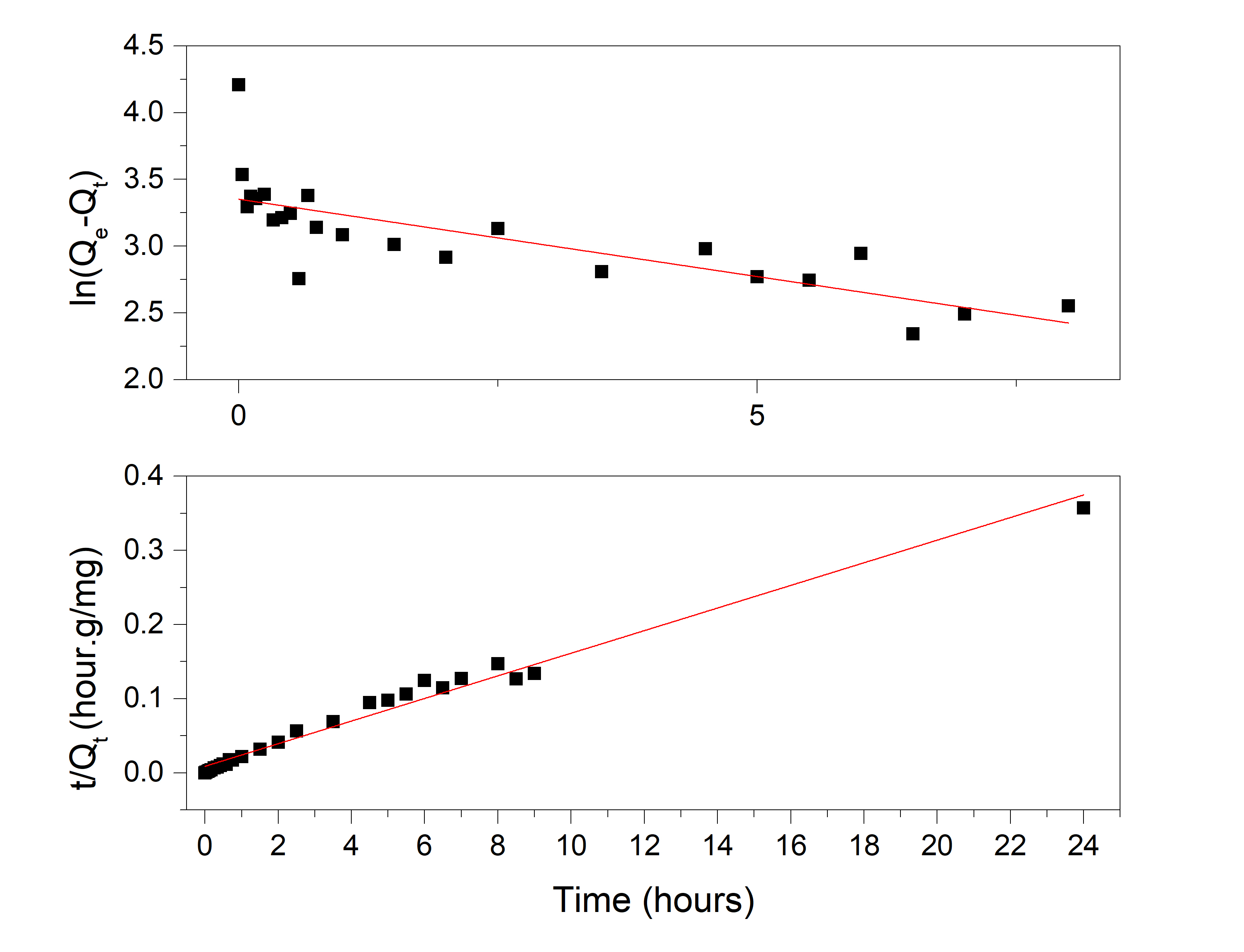

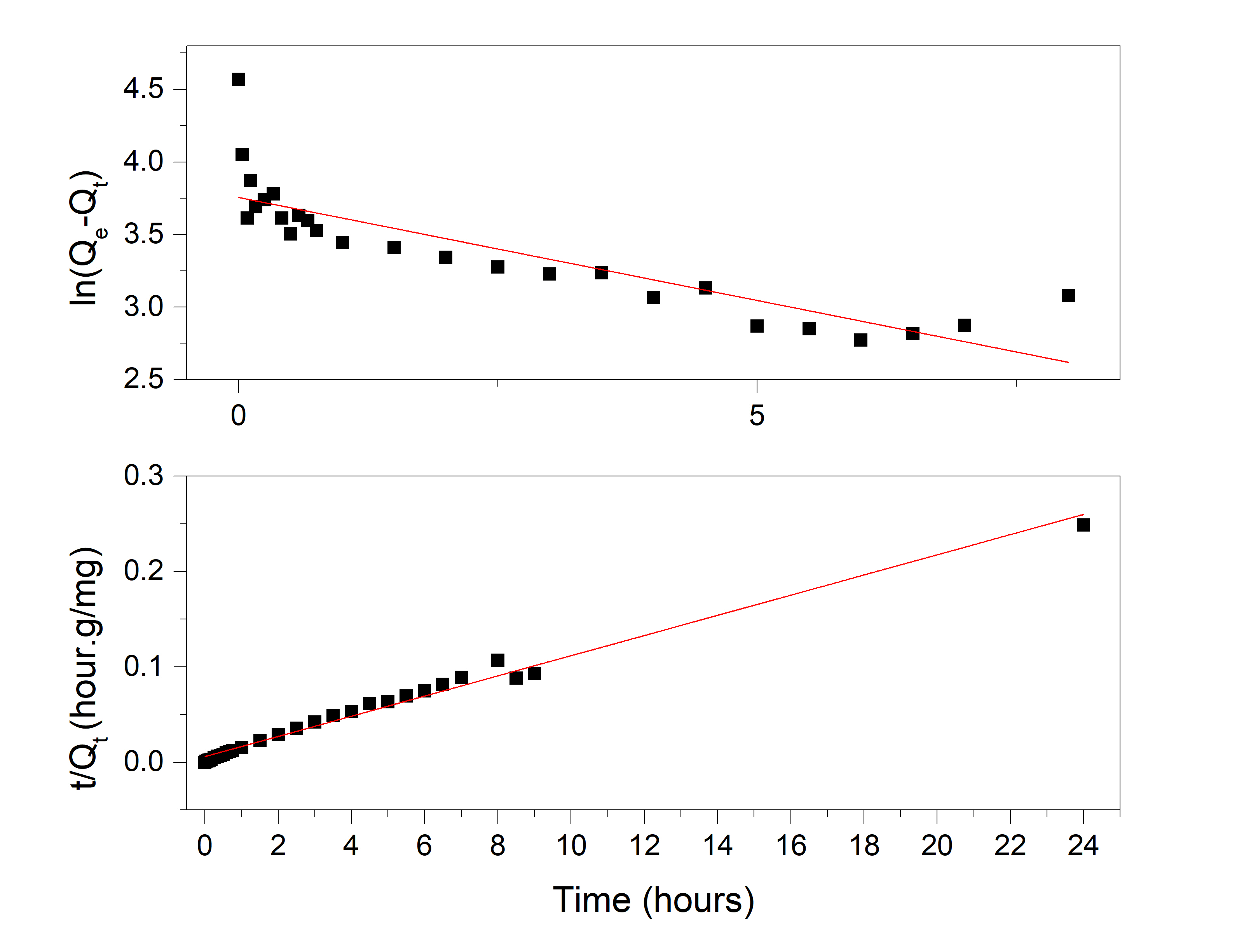


b

a

**Fig.S4** Pseudo first order kinetic model (Top) and Pseudo second order kinetic model (bottom) fitting for A) AMX and B) TC, adsorption onto Ag/AC


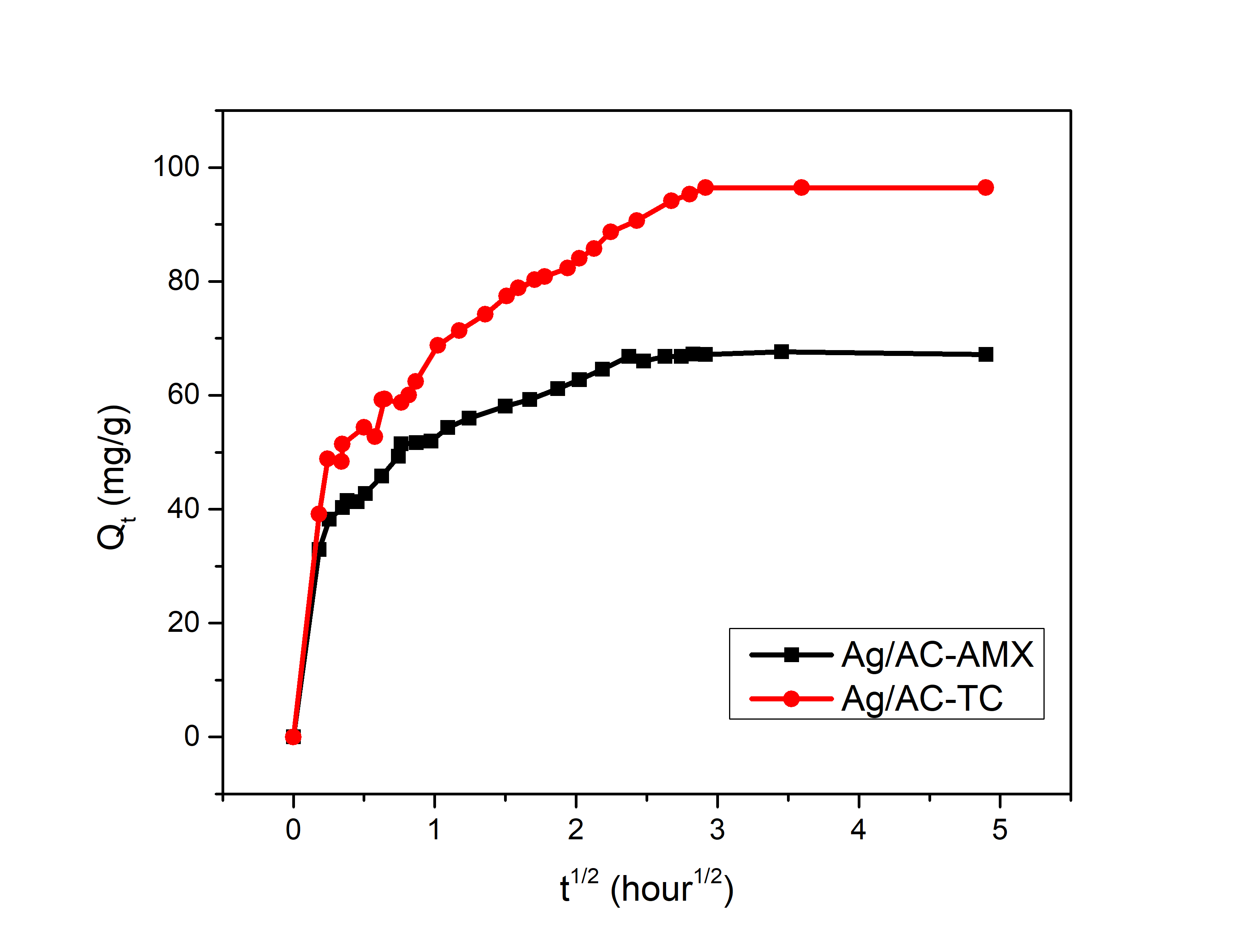


**Fig.S5** Intraparticle diffusion model fitting for AMX and TC adsorption using Ag/AC
